# Bioprospecting saline gradient of a Wildlife Sanctuary for bacterial diversity and antimicrobial activities

**DOI:** 10.1186/s13104-017-2711-9

**Published:** 2017-08-11

**Authors:** Mara DeLuca, Riley King, Mustafa Morsy

**Affiliations:** Department of Biological and Environmental Sciences, University of West Alabama, Livingston, AL 35470 USA

**Keywords:** Antimicrobial producing bacteria, Bacterial diversity, Saline soil

## Abstract

**Objective:**

Antibiotic-resistant bacteria are becoming a global crisis, causing death of thousands of people and significant economic impact. The discovery of novel antibiotics is crucial to saving lives and reducing healthcare costs. To address the antibiotic-resistant crisis, in collaboration the Small World Initiative, which aims to crowdsource novel antibiotic discovery, this study aimed to identify antimicrobial producing bacteria and bacterial diversity in the soil of the Stimpson Wildlife Sanctuary, an inland area with a soil salt gradient.

**Results:**

Approximately 4500 bacterial colonies were screened for antimicrobial activity and roughly 100 bacteria were identified as antimicrobial producers, which belong to Entrococcaceae (74%), Yersiniaceae (19%), and unidentified families (7%). Several bacterial isolates showed production of broad spectrum inhibitory compounds, while others were more specific to certain pathogens. The data obtained from the current study provide a resource for further characterization of the soil bacteria with antimicrobial activity, with an aim to discover novel ones. The study showed no correlation between soil salt level and the presence of bacteria with antimicrobial activities. However, most of the identified antimicrobial producing bacteria do not belong to actinomycetes, the most common phyla of antibiotic producing bacteria and this could potentially lead to the discovery of novel antibiotics.

**Electronic supplementary material:**

The online version of this article (doi:10.1186/s13104-017-2711-9) contains supplementary material, which is available to authorized users.

## Introduction

While the increasing use of antibiotics in medical and agricultural applications has been beneficial, in recent years overuse of broad-spectrum antibiotics combined with the evolution of bacteria has led to the evolution of deadly antibiotic-resistant bacteria [1–4]. The evolution of antibiotic-resistant bacteria is a global crisis causing the death of at least 23,000 people in the United States [5, 6]. Antibiotic-resistant bacteria have a negative economic impact and lead to increased costs associated with the health care of infected individuals. For example, *Clostridium difficile* is a microbe causing extreme gastrointestinal issues with acute infections resulting in death within thirty days of being infected for some 29,000 patients [7].

To combat the antibiotic-resistant bacteria crisis, there is a dire need for discovery and development of novel and effective antimicrobial compounds to treat these infections. Despite this need, major pharmaceutical companies are not investing in novel antibiotic development due to low profitability [8, 9] and other significant obstacles such as the instability of the global public health infrastructure and availability of funding and technology in underdeveloped countries. In addition, there is a need to limit the use of broad-spectrum antibiotics, thus minimizing the development of new superbugs [10].

To address the antibiotic-resistant bacteria crisis and as a part of the Small World Initiative (http://www.smallworldinitiative.org) course, two students aimed to explore the discovery of antimicrobial producing bacteria in the soil of the Stimpson Wildlife Sanctuary (SWS). The SWS has unique soil with many salt springs that allow for the formation of salt gradients ranging from fresh water to water with up to 50 Parts Per Thousand (PPT) Total Dissolved Salts (TDS). Bacteria that inhabit this area represent untapped sources of potentially new bioactive compounds, including antibiotics. This area provides an opportunity not only to discover antibiotic-producing bacteria but also to explore the bacterial diversity in response to salt stress. In this study, a culture-dependent approach was applied to isolate and discover bacteria with antimicrobial activities.

## Main text

### Materials and methods

#### Soil collection site

Samples were collected from the SWS located in Alabama, United States. A permission to collect samples was obtained from the Alabama Department of Conservation and Natural Resources. The SWS is characterized by the presence of a salt gradient, ranging from fresh water to water with up to 50 PPT TDS. Twenty soils and water samples were collected at various locations around SWS into sterilized 50 ml tubes and kept on ice during transportation to the laboratory. The water samples’ TDS was measured on site using HM Digital TDS meter.

#### Bacteria isolation

One gram of soil was suspended into 10 ml 0.85% w/v NaCl, vortexed, spun down, and the supernatant was collected. A serial dilution was made, followed by spreading 100 µl onto Luria–Bertani (LB), tryptic soy, potato dextrose, terrific broth, yeast extract peptone dextrose media with 1.5% w/v agar. All plates were incubated overnight at 37 °C. The number of individual bacterial colonies and number of bacterial phenotypes were recorded. About 4500 bacterial colonies were picked into a 96-deep well plate containing 400 µl LB broth and incubated in 37 °C shaker set at 200 rpm for 24 h.

#### Screening of antimicrobial activity

Bacteria isolated from the soil were tested for antimicrobial activities against safe relatives of the ESKAPE pathogens, six pathogens with growing multidrug resistant virulence [11] and *Salmonella Newport*, a major cause of food poisoning [12]. A liquid culture of a single colony of each ESKAPE relative was grown in 5 ml LB media for 12 h at 34 °C while shaking. Optical Density 600 was adjusted for all ESKAPE relatives to an average of 4 × 10^6^ CFU/ml. To generate a bacterial lawn, 200 µl of liquid ESKAPE cultures were plated onto 150 mm Petri dishes with LB agar. Unknown bacterial strains were laid over ESKAPE plates using a 96-well pin replicator and incubated overnight at 26 °C. After 36 h, antimicrobial activity was assessed by the presence of inhibition zones of the ESKAPE relatives around the unknown bacterial strains.

#### Bacterial molecular identification

Antibiotic-producing bacteria were identified using 16S rRNA sequence. A small portion of every colony was used as a genomic DNA template in the PCR mix. The 16S rRNA gene primers (27 forward and 1492 reverse) were used to amplify and sequence a 1465 bp [13]. PCR products were separated using 1% agarose gel, purified using 5 Prime GelElute Extraction Kit (Prime, Gaithersburg, MD), and then sequenced. DNA sequences were assembled using DNAStar and then searched against the non-redundant DNA sequence available in the NCBI database.

### Results

#### Bacterial isolation

Samples from Cypress Knees, which frequently are submerged in water, demonstrated the lowest number of bacterial colonies (1454 CFU/ml). On the other hand, other samples showed a much higher number of bacterial colonies, with the Tree Log having the maximum number of colony forming units (12,114 CFU/ml), Table (1).

**Table 1:** Number of single bacterial colony isolated from the SWS locations and number and percentage of bacteria with antimicrobial activities

Sample location	CFU (ml)	No. of APB	% of APB
0PPT	8314	3	3.1
14PPT	10,774	9	9.4
20PPT	6246	4	4.2
50PPT	7914	9	9.4
Tree log	12,114	14	14.7
Cypress knee	1454	10	10.5
Total	46,816	49	

Single bacterial colonies were isolated from each sample followed by antimicrobial screening against 4 different ESKAPE relatives

#### Antimicrobial Screening

**Fig. 1:**
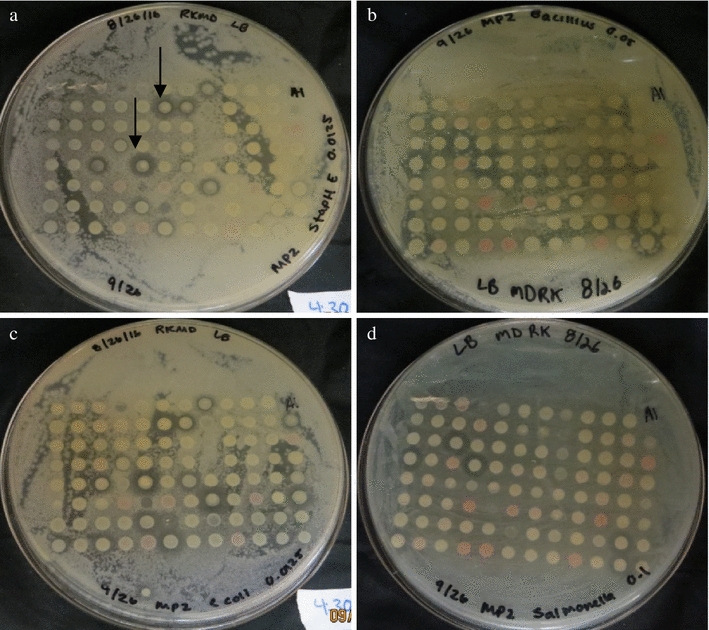
Screening of antimicrobial activity of unknown bacteria against known pathogens via co-cultivation. Antimicrobial screening against **a** *S. epidermidis* **b** *B. subtilis*, **c** *E.* coli and **d** *S. newport*. *Black arrows* indicate antagonistic activity or inhibition zones surrounding some of the unknown soil bacteria

A set of 95 bacterial colonies from each sample site were co-cultured over a lawn of 4 different ESKAPE relatives, safe relatives and the formation of inhibition zones were identified (Fig. 1). The size of the inhibition zone varied based on the antimicrobial activities of these bacteria. We quantified the production of antimicrobial activity by measuring the size of the inhibition zone. Sample 8A isolated from soil near Cypress Knees and identified as *Enterococcus faecalis* showed the highest antibiotic production (Fig. 1). This bacterial strain was effective in inhibiting the growth of *S. newport* and *B. subtilis*. However, sample 12B isolated from Cypress Knees and identified as *E. faecalis* had the lowest inhibition of bacteria (Fig. 1). This strain was effective in inhibiting *B. subtilis* only. Samples obtained from Tree Log and Cypress Knees have the most antimicrobial producing bacteria (14.7 and 10.5%, respectively), while soil near fresh water and 20 PPT salt had the lowest number of antimicrobial producing bacteria (3.1 and 4.2%, respectively). Soil samples near water with 14 PPT and 50 PPT salt showed moderate levels of antimicrobial producing bacteria, about 9% (Table 1).

The specificity of the antimicrobial activities against known ESKAPE relatives was identified (Additional file 1: Table S1). Sample number D9 and H12 were effective in inhibiting four different ESKAPE (*S. epidermidis*, *B. subtilis*, *E. coli* and *S. newport*). We have observed that *S. epidermidis* and *E. coli* were killed by bacteria isolated from any of the sampling sites, which indicates that these bacteria are susceptible to a wide range of antimicrobial compounds. Two antimicrobial producing bacteria were effective only on *E. coli*, which indicates that the inhibitory compound produced by these bacteria has a very narrow effectiveness on various ESKAPE relatives.

#### Bacterial diversity

**Fig. 2:**
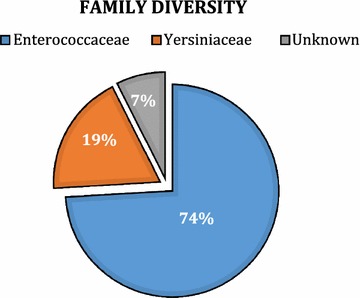
Diversity of antimicrobial compounds producing bacteria based on their family

The overall diversity of the antimicrobial producing bacteria is minimal across all samples (Fig. 2). Most of the antimicrobial compounds producing bacteria belonged to the family Entrococcaceae (74%), followed by Yersiniaceae (19%), and very few were unidentified bacteria (7%). However, among samples of the same nature (Tree Log/Cypress Knees vs. soil samples), a number of unique bacterial strains were isolated (Additional file 3: Table S3). Unique bacterial strains are denoted by asterisks. For example, the Cypress Knees and the Tree Log have 5 and 6 unique bacterial strains, respectively, while other bacteria were common in other samples (Additional file 2: Table S2). Similarly, the different soil samples showed a number of unique bacterial isolates, with soil near fresh water showing the minimum uniqueness (2 isolates), while soil near 50 PPT water showed the number of unique isolates (5 isolates) (Additional file 3: Table S3).

### Discussion

Bacteria that inhabit the soil of environmentally stressed areas such as high salinity, represent a rich source of potential novel antimicrobial compounds producing bacteria. In addition, the diversity of soil bacteria under a stressed environment is usually lower compared to bacteria present in other soil. The Stimpson Wildlife Sanctuary is a unique soil habitat representing high and low salt levels within a relatively short distance. The physical state of this distinct habitat allowed the presence of bacterial strains to adapt to such environments. These bacterial strains most likely maintain specific metabolic pathways that are capable of producing secondary metabolites, including antimicrobial agents against other bacteria. In this study, we used a culture-dependent method to assess bacterial diversity and discover antimicrobial compounds producing bacteria present in the SWS’s soil and organic matter.

A Cypress knee is a distinctive structure formed above the roots of various tree species growing in swamps, which help in oxygenation to the tree’s roots or assist in anchoring the tree in the soft and muddy soil [14]. Therefore, obtaining minimal bacterial diversity from the Cypress knee samples is not a surprise, as most of the associated bacteria are expected to be anaerobic while we focused on isolation of aerobic bacteria. Nutrient and carbon availability and soil moisture have been implicated as factors that may influence microbial community composition [15]; therefore, the number of bacteria obtained from the Tree Log, which is rich in nutrients, was much higher than the Cypress knees. Based on the rRNA sequencing data, Cypress knees’ bacterial community was more diverse and had more unique bacterial isolates compared to the Tree Log. Salinity is the major environmental element that affects soil microbial community, more than extremes of temperature, pH, or physical soil composition [16]. Our data showed higher number of CFUs from soil nearby 50 PPT water; however, there were no specific trends of the number of bacteria and the changes in salt levels. Interestingly, the soil nearby with higher salt levels of 14 PPT and 50 PPT showed the most unique bacterial strains.

Antimicrobial activity testing against the safe relative of the ESKAPE relatives showed various levels of inhibition’s induction and specificity. It has been reported that the antimicrobial compounds are very diverse [17]; therefore it is an expected result to find variation on the level of inhibition and specificity. The identification of 54 bacterial isolates based on the 16S rRNA sequencing assigned them to two different phyla (Proteobacteria and Firmicutes). Interestingly, Actinomycetes, some of the most common soil bacteria that produce 60% of clinically important antibiotics [18], were not identified in our collection. Identification of antimicrobial compounds producing bacteria from phyla other than Actinomycetes is promising; some of these compounds may be novel. The mode of action and the nature of the identified antimicrobial compounds by these bacteria are unknown and its inhibitory effect could be due to production of antibiotics or other chemical production. Overall, most isolates in this study were identified as *E. faecalis* (Additional file 3: Table S3), which is part of the normal microbiota of animals and can cause disease as well [19]. In addition, recovery of *E. faecalis* as the major bacteria could have been due to the methodology of using higher incubation temperature and aerobic conditions.

The Cypress Knee samples have two putative novel and unique bacterial species (sample 7G and 6A) with similarity to unknown bacteria in the Genbank database of less than 100%. Novel and/or known bacterial species represent a unique opportunity to possibly find novel metabolic pathways, including antibiotic production pathways. On the other hand, the Tree Log samples have all been previously identified bacteria, with some isolates having less than 100% match in the database which may indicate new bacterial strains.

Some of the bacterial strains with broad spectrum inhibitory compounds may be promising to identify the chemical nature of these compounds and can be used to treat infections of *S. epidermidis*, *B. subtilis*, *E. coli* and *S. newport*. Examples of these antibiotic-producing bacteria are *Serratia* sp. SH-AB-1 and *S. marcescens* [20]. Recent studies have characterized and sequenced the genome of specific strain of *S. marcescens*, which is capable of producing multiple antibiotics [21, 22]. Interestingly, several *Serratia* sp. are also antibiotic-resistant bacteria [23]. Overall, antibiotic-producing bacteria that are capable of inhibiting growth of both gram positive and negative bacteria (i.e. *S. epidermidis* and *E. coli*) were found in all sample sources. Antimicrobial compounds producing bacteria (isolated from Tree Log) were effective only on gram-negative (*E. coli*).

### Strength and limitations

This is the first study that explored the bacterial diversity of an inland saline gradient (SWS) and identified antimicrobial producing bacteria. This study provided opportunities for undergraduates to perform authentic research and have a sense of ownership of their project. The major limitation of this study is the lack of comprehensive coverage of bacterial diversity in the soil, lack of physiological characterization of these bacteria, and the need for chemical characterization of the produced inhibitory compounds.

## Limitations

BMC Research Notes considers scientifically valid manuscripts irrespective of the interest of a study or its likely impact. In order to ensure submissions to BMC Research Notes are of maximum benefit to the research community, authors should clearly state the limitations of their work.

## Additional files

**Additional file 1: Table S1.** Pathogen Specificity of unknown bacteria against different pathogen. Inhibitory effects were varied based on the pathogen, some bacteria were more specific to particular pathogen and others have a wide spectrum inhibitory effect.

**Additional file 2: Table S2.** Identity of Bacteria isolated from the SWS decomposed and plant materials. Bacterial strains were identified based on the 16S rRNA sequence.

**Additional file 3: Table S3.** Identity of bacteria isolated from the Stimpson Wild Life Sanctuary’s soils with various salt levels. Bacterial strains were identified based on the 16S rRNA sequence.
