# Supplementary material for: Bioprospecting saline gradient of a Wildlife Sanctuary for bacterial diversity and antimicrobial activities
Source: BMC Res Notes. 2017 Aug 11;10:397. doi: 10.1186/s13104-017-2711-9 (PMC5553665; doi:10.1186/s13104-017-2711-9)
Supplement: Supplementary file 1 — Additional file 1: Table S1. Pathogen Specificity of unknown bacteria against different pathogen. Inhibitory effects were varied based on the pathogen, some bacteria were more specific to particular pathogen and others have a wide spectrum inhibitory effect. [file 13104_2017_2711_MOESM1_ESM.pdf]

Additional file 1: Table S1. Pathogen Specificity of unknown bacteria against different pathogen. Inhibitory effects were varied based on the pathogen, some bacteria were more specific to particular pathogen and others have a wide spectrum inhibitory effect.

Source	Sample ID	Pathogen Specificity
**0 ppt**		
	G2	*B. subtilis* and *S. newport* Sp.
	H2	*B. subtilis*, *S. newport* and *E. coli*
	B7	*S. aureus* and *E. coli*
**20 ppt**		
	B6	*S. aureus* and *E. coli*
	E8	*S. aureus* and *E. coli*
	F1	*S. aureus* and *E. coli*
	G8	*S. aureus* and *E. coli*
	H7	*S. aureus* and *E. coli*
**50 ppt**		
	D9	*S. aureus, B. subtilis*, *E. coli* and *S. newport*
**Cypress Knees**		
	D12	*B. subtilis* and *S. newport*
	H12	*S. aureus*, *B. subtilis*, *E. coli* and *S. newport*
	F5	*S. aureus*, *E. coli* and *B. subtilis*
**Tree Log**		
	B1	*E. coli*
	H10	*E. coli*
	A5	*S. aureus* and *E. coli*
	E6	*S. aureus* and *E. coli*
	E10	*S. aureus* and *E. coli*
	C10	*S. aureus*, *B. subtilis* and *S. newport*
