# Supplementary material for: Bioprospecting saline gradient of a Wildlife Sanctuary for bacterial diversity and antimicrobial activities
Source: BMC Res Notes. 2017 Aug 11;10:397. doi: 10.1186/s13104-017-2711-9 (PMC5553665; doi:10.1186/s13104-017-2711-9)
Supplement: Supplementary file 2 — Additional file 2: Table S2. Identity of Bacteria isolated from the SWS decomposed and plant materials. Bacterial strains were identified based on the 16S rRNA sequence. [file 13104_2017_2711_MOESM2_ESM.pdf]

Additional file 2: Table S2. Identity of Bacteria isolated from the SWS decomposed and plant materials. Bacterial strains were identified based on the 16S rRNA sequence.

Sample Name	GenBank ID	Description	Query cover	e value
**Cypress Knees**				
2B/6C/7H/8A	gb|KU937390.1|	*Enterococcus faecalis* strain GX27	99%	0
7F	gb|KT343158.1|	*Enterococcus faecalis* strain JF85	100%	0
9E	gb|KC335216.1|	*Serratia marcescens* strain LB21	99%	0
12B	gb|KC335216.1|	*Serratia marcescens* strain LB21	99%	0
*12A	gb|AY514434.1|	*Serratia marcescens* strain N2.4	99%	0
*7G	gb|JX006713.1|	Bacterium NLAE-zl-H507	100%	0
*8E	gb|KC213479.1|	*Enterococcus durans* strain GM19	99%	0
*9G	gb|KJ726743.1|	*Enterococcus faecalis* strain NIOT-COMAPS02	99%	0
*6A	gb|JX006721.1|	Bacterium NLAE-zl-H515	99%	0
**Tree Log**				
4B/4E	gb|KU937390.1|	*Enterococcus faecalis* strain GX27	99%	0
8C	gb|JX006717.1|	*Enterococcus* sp. Le5-1a	100%	0
10A	gb|KT343158.1|	*Enterococcus faecalis* strain JF85	100%	0
6H	gb|KU937390.1|	*Enterococcus faecalis* strain GX27	99%	0
11B	gb|KC335216.1|	*Serratia marcescens* strain LB21	99%	0
5C	gb|JX006717.1|	*Enterococcus* sp. Le5-1a	100%	0
*4D	gb|KM207818.1|	*Enterococcus faecalis* strain FT522	99%	0
*4G	gb|GQ337884.1|	*Enterococcus faecalis* strain KLDS4.0341	99%	0
*6D	gb|EU794735.1|	*Enterococcus* sp. Le5-1a	100%	0
*4A	gb|HQ831381.1|	*Enterococcus faecalis* strain Na15	99%	0
*2C	gb|KY438200.1|	*Enterococcus faecalis* strain AF12	99%	0
*11A	gb|GU458280.1|	*Serratia sp.* PT1B	99%	0
