# Supplementary material for: Bioprospecting saline gradient of a Wildlife Sanctuary for bacterial diversity and antimicrobial activities
Source: BMC Res Notes. 2017 Aug 11;10:397. doi: 10.1186/s13104-017-2711-9 (PMC5553665; doi:10.1186/s13104-017-2711-9)
Supplement: Supplementary file 3 — Additional file 3: Table S3. Identity of bacteria isolated from the Stimpson Wild Life Sanctuary’s soils with various salt levels. Bacterial strains were identified based on the 16S rRNA sequence. [file 13104_2017_2711_MOESM3_ESM.pdf]

Additional file 3: Table S3. Identity of bacteria isolated from the Stimpson Wild Life Sanctuary's soils with various salt levels. Bacterial strains were identified based on the 16S rRNA sequence.

**Sample Name**	**GenBank ID**	**Description**	**Query cover**	**e value**
**0 PPT**				
8G/3E	gb|KU937390.1|	*Enterococcus faecalis* strain GX27	99%	0
8B	gb|GQ337884.1|	*Enterococcus faecalis* strain KLDS4.0341	99%	0
*6D/12G	gb|EU794735.1|	*Enterococcus sp.* Le5-1a	100%	0
*3F	gb|KC213478.1|	*Enterococcus durans* strain GM18	100%	0
**14ppt**				
8F/1H	gb|KU937390.1|	*Enterococcus faecalis* strain GX27	99%	0
1G/8D	gb|GQ337884.1|	*Enterococcus faecalis* strain KLDS4.0341	99%	0
7C	gb|KU937390.1|	*Enterococcus faecalis* strain GX27	99%	0
7A	gb|KT343158.1|	*Enterococcus faecalis* strain JF85	100%	0
3B	gb|EU708623.1|	*Enterococcus faecalis* strain XR7	99%	0
9D	gb|KP241784.1|	*Serratia sp.* SH-AB-1	99%	0
*2E	gb|HQ831381.1|	*Enterococcus faecalis* strain Na15	99%	0
*2F	gb|HQ184922.1|	*Enterococcus faecalis* strain FUA3334	99%	0
*7B	gb|JX006538.1|	Bacterium NLAE-zl-H323	99%	0
**20ppt**				
*6B/8h/10F	gb|KT343158.1|	*Enterococcus faecalis* strain JF85	100%	0
**50ppt**				
5G/5H	gb|EU708623.1|	*Enterococcus faecalis* strain XR7	99%	0
9C/12C	gb|KP241784.1|	*Serratia sp.* SH-AB-1	99%	0
5E	gb|KX752874.1|	*Enterococcus hirae* strain SNNU0253	99%	0
*5D	gb|JX006721.1|	Bacterium NLAE-zl-H515	99%	0
*3G	gb|HQ831431.1|	*Enterococcus faecalis* strain TLME3	99%	0
*5F	gb|JF947360.1|	*Morganella morganii* strain 2113	98%	0
*9B	gb|KT894728.1|	*Serratia marcescens* strain JND-KHCo-24B	99%	0
*10H	gb|AF513469.1|	Enterobacteriaceae bacterium PH31	99%	0
